# Knockdown Resistance Mutations in the Voltage-Gated Sodium Channel of *Aedes aegypti* (Diptera: Culicidae) in Myanmar

**DOI:** 10.3390/insects13040322

**Published:** 2022-03-25

**Authors:** Haung Naw, Tuấn Cường Võ, Hương Giang Lê, Jung-Mi Kang, Yi Yi Mya, Moe Kyaw Myint, Tong-Soo Kim, Ho-Joon Shin, Byoung-Kuk Na

**Affiliations:** 1 Department of Parasitology and Tropical Medicine, Institute of Health Sciences, Gyeongsang National University College of Medicine, Jinju 52727, Korea; haungnaw23@gmail.com (H.N.); vtcuong241@gmail.com (T.C.V.); gianglee291994@gmail.com (H.G.L.); gjm9951001@hanmail.net (J.-M.K.); 2 Department of Convergence Medical Science, Gyeongsang National University, Jinju 52727, Korea; 3 Department of Medical Research Pyin Oo Lwin Branch, Pyin Oo Lwin, Myanmar; yimya.dmrum@gmail.com (Y.Y.M.); dr.myintmoekyaw@gmail.com (M.K.M.); 4 Department of Tropical Medicine, Inha Research Institute for Medical Sciences, Inha University College of Medicine, Incheon 22212, Korea; tongsookim@inha.ac.kr; 5 Department of Microbiology, Ajou University College of Medicine, Suwon 16499, Korea; hjshin@ajou.ac.kr

**Keywords:** *Aedes aegypti*, voltage-gated sodium channel, knockdown resistance, Myanmar

## Abstract

**Simple Summary:**

Knockdown resistance (*kdr*) mutations in the voltage-gated sodium channel (VGSC) of mosquitoes confer resistance to pyrethroid insecticides. Analysis of *kdr* mutations in *Aedes aegypti* mosquitoes collected from five different townships in the Mandalay area, Myanmar, revealed high levels of validated *kdr* mutations in domains II and III of *vgsc*. Moreover, high frequencies of concurrent *kdr* mutations were also detected. The results of this study suggest that *kdr* mutations associated with pyrethroid resistance are widespread in the *Ae. aegypti* population of the study area. Our results provide a valuable molecular basis to understand the pyrethroid resistance status of the *Ae. aegypti* population in the area and underscore the need for an effective vector control program in Myanmar.

**Abstract:**

*Aedes aegypti* is an important mosquito vector transmitting diverse arboviral diseases in Myanmar. Pyrethroid insecticides have been widely used in Myanmar as the key mosquito control measure, but the efforts are constrained by increasing resistance. Knockdown resistance (*kdr*) mutations in the voltage-gated sodium channel (VGSC) are related to pyrethroid resistance in *Ae. aegypti*. We analyzed the patterns and distributions of the *kdr* mutations in *Ae. aegypti* in the Mandalay area of Myanmar. The segment 6 regions of domains II and III of *vgsc* were separately amplified from individual *Ae. aegypti* genomic DNA via polymerase chain reaction. The amplified gene fragments were sequenced. High proportions of three major *kdr* mutations, including S989P (54.8%), V1016G (73.6%), and F1534C (69.5%), were detected in the *vgsc* of *Ae. aegypti* from all studied areas. Other *kdr* mutations, T1520I and F1534L, were also found. These *kdr* mutations represent 11 distinct haplotypes of the *vgsc* population. The S989P/V1016G/F1534C was the most prevalent, followed by S989P/V1016V and V1016G/F1534C. A quadruple mutation, S989P/V1016G/T1520I/F1534C, was also identified. High frequencies of concurrent *kdr* mutations were observed in *vgsc* of Myanmar *Ae. aegypti*, suggesting a high level of pyrethroid resistance in the population. These findings underscore the need for an effective vector control program in Myanmar.

## 1. Introduction

*Aedes aegypti* is the primary mosquito vector transmitting diverse arboviral pathogens such as dengue, chikungunya, Zika, and yellow fever viruses to humans in wide geographical areas [1]. This anthropophilic mosquito species originated in Africa and rapidly spread worldwide by successfully adapting to artificial environments [2]. Their ability to thrive in urban environments and to travel long distances via transportation strengthens their vectorial competence. Rapid urbanization and climate change also accelerated the widespread distribution of *Ae. aegypti* in tropical and subtropical areas [3].

*Ae. aegypti* has been mainly controlled by environmental management and the use of insecticides such as pyrethroids, which are widely employed globally due to their photostability, low mammalian toxicity, high efficacy, and rapid paralysis or “knockdown” [4]. Pyrethroid insecticides target the voltage-gated sodium channel (VGSC) in insect neurons and alter the action potential, thereby resulting in paralysis and death of mosquitoes [4]. However, the extensive use of pyrethroids has led to the emergence of insecticide resistance in *Ae. aegypti* populations and global outbreaks of arboviral diseases [5,6]. VGSC is a transmembrane protein found in the mosquito’s nerve cell membranes. This protein consists of four homologous domains (domains I–IV), each of which comprises six hydrophobic subunits, segments 1–6 [7]. The helical segments 5 and 6 of domain II along with the helical segment 6 of domain III form a hydrophobic pocket for binding with insecticidal compounds [8]. However, the indiscriminate use of pyrethroids induces resistance mediated by knockdown resistance (*kdr*) mutations in the *vgsc*. The *kdr* mutations induce structural changes in the VGSC, which prevent the binding of pyrethroids to the target site, resulting in insecticidal resistance [9,10,11]. Until now, diverse *kdr* mutations contributing to pyrethroid resistance have been detected in the global *Ae. aegypti* population. These mutations, including V410L, G923V, L982W, S989P, I1011M/V, L1014F/S, V1016G/I, T1520I, F1534C, and D1763Y, generate diverse and continent-specific *kdr* genotypes in the global *Ae. aegypti* populations [9,12,13,14,15,16].

*Ae. aegypti* is the primary vector transmitting dengue fever in Myanmar. The warm and humid climate throughout the year and unsanitary environments provide ideal breeding conditions for the mosquito species, resulting in large numbers of annual dengue fever cases in the country. More than 20,000 annual cases have been reported in Myanmar during the last decade [17,18]. Pyrethroid insecticides have been widely used as the principal control measure for *Ae. aegypti* in Myanmar; however, the extensive and indiscreet use of the insecticides has increased concerns regarding insecticide resistance in the *Ae. aegypti* population, which threatens effective control of the mosquito species. In this respect, understanding the insecticide resistance status is imperative to implement appropriate guidelines or alternative strategies for *Ae. aegypti* control in Myanmar.

Molecular approaches to analyze *kdr* mutations in *vgsc* of *Ae. aegypti* are valid tools to monitor insecticide resistance [9,12,15]. Molecular studies revealed a substantial number of *kdr* mutations associated with insecticide resistance in the Myanmar *Ae. aegypti* population [19,20,21]. In this study, we analyzed the patterns and prevalence of *kdr* mutations in *vgsc* of *Ae. aegypti* collected in the Mandalay area, Myanmar, in order to extend our knowledge of the insecticide resistance status of the mosquito population in the country.

## 2. Materials and Methods

### 2.1. Mosquito Collection

**Figure 1:**
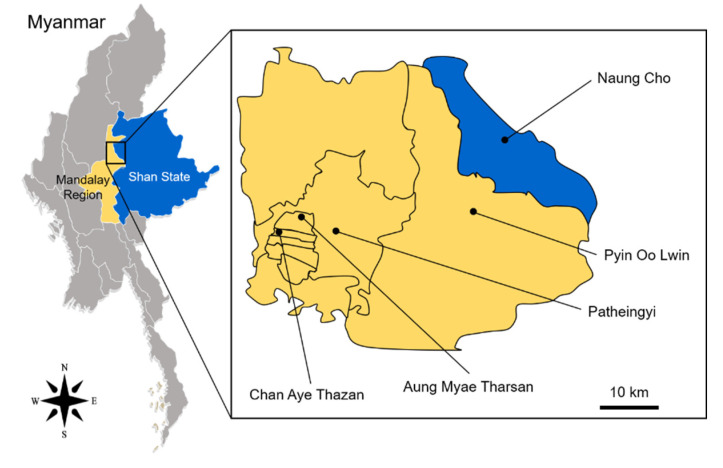
Map of the mosquito collection sites. *Aedes aegypti* larvae and pupae were collected at 4 townships in Mandalay Region [Patheingyi (21°57′44.7″ N, 96°24′20.9″ E), Pyin Oo Lwin (21°59′25.9″ N, 96°04′13.0″ E), Chan Aye Thazan (21°57′44.7″ N, 96°24′20.9″ E), and Aung Myae Tharsan (21°58′39.6″ N, 96°05′54.3″ E)] and 1 township in Shan State [Naung Cho (22°19′48.7″ N, 96°47′24.0″ E)]. Yellow and blue colors represent Mandalay Region and Shan State, respectively. Closed black circles indicated the collection sites of the mosquito samples.

*Aedes aegypti* larvae and pupae were collected from 5 different townships: Patheingyi (21°57′44″ N, 96°24′20.9″ E), Pyin Oo Lwin (21°59′25.9″ N, 96°04′13.0″ E), Chan Aye Thazan (21°57′44.7″ N, 96°24′20.9″ E), and Aung Myae Tharsan (21°58′39.6″ N, 96°05′54.3″ E) in the Mandalay Region, and Naung Cho (22°19′48.7″ N, 96°47′24.0″ E) in Shan State (Figure 1). The mosquito samples were collected from artificial water containers, such as discarded tires, metal drums, water storage clay pots, enamel jars, concrete water storage tanks, or flower vases around households at different locations of each township during July to October 2019 at the peak of mosquito breeding and dengue fever transmission. The collected mosquito samples were transferred to clean water containers, transported to the Department of Medical Research Pyin Oo Lwin Branch, and reared to adults in the laboratory insectary at a temperature of 25 ± 2 °C and humidity of 80 ± 10%. Adult *Ae. aegypti* mosquitoes were further confirmed using standard mosquito classification methods [22].

### 2.2. DNA Extraction and Gene Amplification

The mosquito DNA was extracted from 411 *Ae. aegypti* mosquitoes individually using a Tissue Genomic DNA Extraction Kit (Bioneer, Daejeon, Korea) following the manufacturer’s instructions. Fragments of the *vgsc* flanking segment 6 of domain II (DII-S6) and segment 6 of domain III (DIII-S6) were amplified via polymerase chain reaction (PCR) with specific primer sets: AaNavF20 (5′-ACAATGTGGATCGCTTCCC-3′) and AaNavR21 (5′-TGGACAAAAGCAAGGCTAAG-3′) for DII-S6, and AaNavEx31P (5′-TCGCGGGAG-GTAAGTTATTG-3′) and AaNavEx31Q (5′-GTTGATGTGCGATGGAAATG-3′) for DIII-S6 [21,23]. The thermal cycling conditions were initial denaturing at 95 °C for 10 min, followed by 35 cycles of denaturation at 95 °C for 30 s, annealing at 56 °C for 30 s and extension at 72 °C for 30 s, and the final extension at 72 °C for 5 min. Each PCR product was analyzed with 1.2% agarose gel, purified from the gel, and ligated into the T&A cloning vector (Real Biotech Corporation, Banqiao City, Taiwan). Each ligation mixture was transformed into *Escherichia coli* DH5α competent cells (Real Biotech Corporation) and positive clones with correct inserts were selected with colony PCR. The nucleotide sequences of the cloned gene fragments were analyzed via Sanger DNA sequencing (Macrogen, Daejeon, Korea). To verify the sequence accuracy of the cloned gene fragments, plasmids from at least two to three individual clones from each mosquito sample were sequenced bidirectionally.

### 2.3. Sequence Analysis

The nucleotide and deduced amino acid sequences of *vgsc* DII-S6 and DIII-S6 were analyzed using Editseq and Seqman programs in the DNASTAR package (DNASTAR, Madison, WI, USA). The amino acid polymorphisms in the sequences were detected by comparing them with the reference sequence of *Ae. aegypti vgsc* (GenBank accession number: KY747529.1). The nucleotide sequences analyzed in this study were deposited at GenBank under the accession numbers OL338546–OL338939 for DII-S6 and OL338940–OL339333 for DIII-S6.

## 3. Results

### 3.1. kdr Mutations in vgsc of Myanmar Ae. aegypti

**Figure 2:**
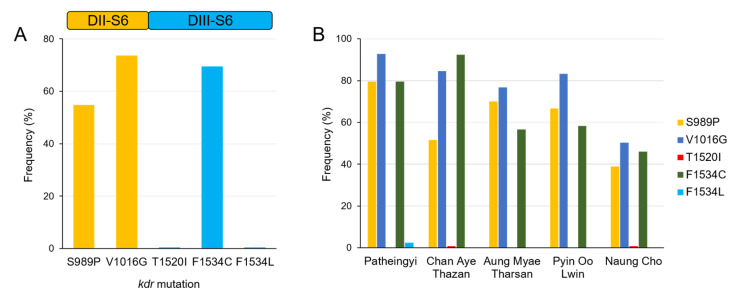
Frequencies of major *kdr* mutations associated with pyrethroid resistance in *Ae. aegypti* populations collected in Mandalay area, Myanmar. (**A**) Frequencies of major *kdr* mutations detected in *vgsc* DII-S6 and DIII-S6 of Myanmar *Ae. aegypti* populations. (**B**) Frequencies of major *kdr* mutations detected in Myanmar *Ae. aegypti* populations collected at 5 townships, Mandalay area, Myanmar.

PCR of DII-S6 and DIII-S6 regions of *vgsc* from 411 Myanmar *Ae. aegypti* mosquitoes resulted in successful amplification of both fragments in 394 mosquitoes. However, both gene fragments were not successfully amplified in five samples, or only one of the two gene fragments was amplified in 12 samples, and thus excluded in further analysis. Due to differences in the number of mosquito samples collected in each township, the number of sequences obtained from each township varied. Most of the sequences originated from *Ae. aegypti* collected in Naung Cho (139, 35.3%) and Chan Aye Thazan (130, 33.0%), followed by Patheingyi (83, 21.1%), Aung Myae Tharsan (30, 7.6%), and Pyin Oo Lwin (12, 3.0%). Four major validated *kdr* mutations, S989P and V1016G in DII-S6, and T1520I and F1534C in DIII-S6, were identified in Myanmar *Ae. aegypti* (Figure 2A). Interestingly, F1534L was also found. Among these, V1016G was predominant (290/394, 73.6%) followed by F1534C (274/394, 69.5%) and S989P (216/394, 54.8%). Meanwhile, T1520I and F1534L were detected with lower frequencies of 0.5%. These mutations showed similar, but not identical, distribution patterns between and among *Ae. aegypti* populations collected from different townships (Figure 2B). Overall frequencies of V1016G and F1534C were relatively high, ranging from 50.4% to 92.8% in *Ae. aegypti* populations from all five townships. S989P was also detected with a high frequency in *Ae. aegypti* populations from Patheingyi (79.5%), Aung Myae Tharsan (70.0%), Pyin Oo Lwin (66.7%), Chan Aye Thazan (51.5%), and Naung Cho (38.9%). A low frequency (0.8%) of T1520I was identified only in *Ae. aegypti* from Chan Aye Thazan and Naung Cho. F1534L was also detected in two mosquito samples (2.4%) from Patheingyi. In addition to these major mutations, diverse minor mutations were also detected in two to five mosquito samples (Table 1). However, F1020S in DII-S6 was detected in 50 *Ae. aegypti* (12.7%) mosquitoes, mainly from Chan Aye Thazan and Naung Cho. E1553G in DIII-S6 was also identified in 10 mosquito samples (2.5%) from Chan Aye Thazan and Aung Myae Tharsan.

### 3.2. vgsc Haplotypes in Myanmar Ae. aegypti

**Figure 3:**
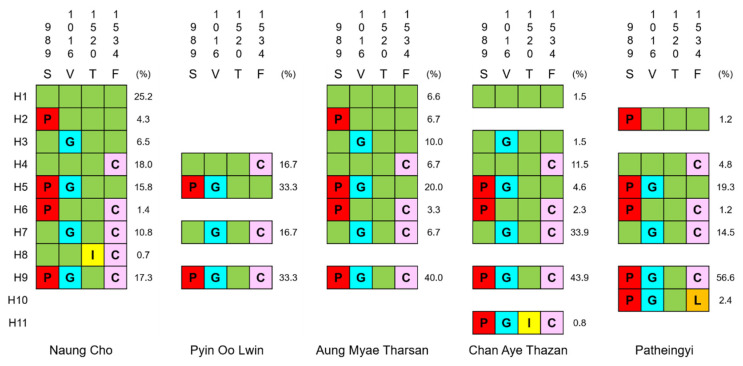
Patterns and frequencies of *vgsc* haplotypes based on major *kdr* mutations in *Ae. aegypti vgsc* from 5 study sites. The wild type amino acids are the column rows and colored boxes indicate mutations. Single or concurrent *kdr* mutations in the *vgsc* DII-S6 and DIII-S6 generated 11 distinct haplotypes of the gene. Patterns and frequencies of each haplotype differed by study site.

Based on the overall patterns and distributions of major *kdr* mutations identified in DII-S6 and DIII-S6 of each *vgsc* sequence, the *Ae. aegypti vgsc* sequences from Myanmar were clustered into 11 different haplotypes, H1–H11 (Figure 3). These haplotypes were not equally distributed in the *Ae. aegypti* population from each township. The haplotypes identified in the mosquitoes collected from different townships were also distinct. *Ae. aegypti* from Naung Cho, Chan Aye Thazan, Aung Myae Tharsan, and Patheingyi showed greater haplotype diversity than that of Pyin Oo Lwin. H1, the wild-type haplotype, was found only in the mosquito samples obtained from Naung Cho, Aung Myae Tharsan, and Chan Aye Thazan. Among the 10 haplotypes carrying *kdr* mutations, seven haplotypes, i.e., excluding H2, H3, and H4, harbored at least two different *kdr* mutations. Four haplotypes, H4, H5, H7, and H9, were generally detected in the *Ae. aegypti* population from all five townships. In particular, H9 (S989P/V1016G/F1534C) was the most predominant haplotype in *Ae. aegypti* from Pyin Oo Lin, Aung Myae Tharsan, Chan Aye Thazan, and Patheingyi. H5 (S989P/V1016G) and H7 (V1016G/F1534C) were also prevalent in the population. Furthermore, H8 (T1520I/F1534C), H10 (S989P/V1016G/F1534L), and H11 (S989P/V1016G/ T1520I/F1534C) were unique to *Ae. aegypti* from Naung Cho, Patheingyi, and Chan Aye Thazan, respectively.

## 4. Discussion

Diverse *kdr* mutations associated with pyrethroid resistance have been identified globally in the *Ae. aegypti* population [9,12,13,14,15,16]. Among these mutations, S989P, V1016G, and F1534C are the most common mutations found in Asian *Ae. aegypti* populations [15,19,21,24,25,26,27,28]. In our previous study, we analyzed the overall profiles of *kdr* mutations in *vgsc* of *Ae. aegypti* collected from the Mandalay area of Myanmar and found substantial levels of potent insecticide resistance in the population [21]. However, our previous study was a pooled analysis of *Ae. aegypti* samples, which prevented accurate determination of the frequency and pattern of *kdr* mutations in individual mosquitos to map insecticide resistance. In the present study, we analyzed the patterns and prevalence of *kdr* mutations in the *vgsc* of individual *Ae. aegypti* in the Mandalay area to investigate the insecticide resistance status of the *Ae. aegypti* population in the area. The three major Asian-type *kdr* mutations strongly linked to pyrethroid resistance, S989P, V1016G, and F1534C, were found in *Ae. aegypti* analyzed in this study with high frequencies: S989P (54.8%), V1016G (73.6%), and F1534C (69.5%). High frequencies of these mutations were also reported in our previous study using pooled *Ae. aegypti* samples collected from the Mandalay area in 2017, but with slightly different frequencies of S989P (68.6%), V1016G (73.5%), and F1534C (40.1%) [21]. High levels of S989P (78.8%), V1016G (84.4%), and F1534C (23.2%) were also reported in the *Ae. aegypti* population from Yangon, Myanmar [19]. These mutations were also routinely found in neighboring countries including India, Laos, and Thailand, but with different frequencies [15,24,25,26,27,28]. T1520I detected in *Ae. aegypti* in this study was also a recently identified *vgsc* mutation in *Ae. aegypti* from India, Pakistan, and China [29,30,31], and most recently in Myanmar *Ae. aegypti* [21]. T1520I alone does not confer pyrethroid or DDT resistance, but when combined with F1534C induces high levels of pyrethroid resistance [29,30]. Therefore, T1520I has been selected in populations with background *kdr* mutation F1534C established in the field [30]. Consistent with previous studies [29,30,31], a strong association of T1520I with F1534C was also observed in the Myanmar *Ae. aegypti* population analyzed in this study. F1534L is another recently identified mutation in Indian *Ae. aegypti* and is likely to be genetically associated with permethrin resistance [32]. This mutation associated with deltamethrin resistance has also been reported in *Ae. albopictus* [33,34]. The F1534L identified in Myanmar *Ae. aegypti* was also strongly linked to both S989P and V1016G in Myanmar *Ae. aegypti*. Interestingly, a substantial frequency (12.7%) of F1020S in DII-S6 was detected in *Ae. aegypti* population from all five study sites, especially Naung Cho and Chan Aye Thazan. This mutation was also identified in our previous study, but its frequency was not high in the mosquitoes collected from the Mandalay area in 2017 [21]. The association of F1020S with pyrethroid resistance is still unclear; however, the increasing frequency of this mutation in the Myanmar *Ae. aegypti* population suggests the need for additional studies to investigate its potent association with pyrethroid resistance.

Concurrent *kdr* mutations involving DII-S6 and DIII-S6 of *vgsc* confer high pyrethroid resistance in *Ae. aegypti* [20,29,30,31]. Diverse haplotypes of concurrent *kdr* mutations were reported in *Ae. aegypti* from neighboring countries including India [29], Thailand [26,35,36], China [37], Laos [28], Pakistan [31], Indonesia [38], and Malaysia [27]. High frequencies of concurrent *kdr* mutations were also detected in the *Ae. aegypti* population analyzed in this study. Seven haplotypes of *vgsc* found in the Myanmar *Ae. aegypti* population harbored co-occurrent *kdr* mutations. H9 (S989P/V1016G/F1534C) was the most predominant followed by H5 (S989P/V1016V) and H7 (V1016G/F1534C). Haplotypes carrying S989P/F1534C (H6), T1520I/F1534C (H8), S989P/V1016G/F1534L (H10), and S989P/V1016G/T1520I/F1534C (H11) were also identified, but their frequencies were not high. Similar patterns of complex and concurrent *kdr* mutations were also detected in our previous study [21]. In addition to these validated *kdr* mutations in DII-S6 and DIII-S6, minor mutations, including F1020S and E1553G, were also detected in *Ae. aegypti* from the Mandalay area, and many of them were concurrent with the major *kdr* mutations. Additional studies are also needed to investigate the mutations contributing to pyrethroid resistance in the mosquito population, despite their generally low frequencies.

## 5. Conclusions

This study clearly suggests that *kdr* mutations associated with pyrethroid resistance are widespread in the *Ae. aegypti* population of the Mandalay area, Myanmar. Continued strong pressure for pyrethroid resistance is driving the mutational changes. Our results also provide a valuable molecular basis to understand the pyrethroid resistance status of the *Ae. aegypti* population in the area. High levels of concurrent *kdr* mutations and the appearance of potent novel mutations suggest the need to investigate the pyrethroid resistance status and underlying resistance mechanisms in the *Ae. aegypti* population in the study area. Accurate assessment of the pyrethroid resistance status in the area requires larger numbers of *Ae. aegypti* samples from diverse sites. These efforts are highly relevant to the *Ae. aegypti* control program in Myanmar for prompt detection of pyrethroid resistance, in addition to updated *Ae. aegypti* control strategies in the country.

## Figures and Tables

**Table 1:** Minor mutations found in *vgsc* DII-S6 and DIII-S6 of Myanmar *Ae. aegypti*.

Domain	Mutation	Patheingyi (*n* = 83)	Chan Aye Thazan (*n* = 130)	Aung Myae Tharsan (*n* = 30)	Pyin Oo Lwin (*n* = 12)	Naung Cho (*n* = 139)	Total (*n* = 394)
DII-S6	D960G		1	1			2
	W966R		2				2
	N967D		1		1		2
	M972V		2				2
	M972I					2	2
	I977T			1		2	3
	E985G		2				2
	I987N			2			2
	W991R	1				1	2
	D992N		2				2
	M994V		2				2
	D998G	2					2
	P1003S			2			2
	F1004I		2				2
	F1020S	4	20	2	2	22	50
DIII-S6	V1512A				2		2
	K1514E					5	5
	M1524V	1	2				3
	L1526P	1				1	2
	Y1527F		1			2	3
	Y1527C	4					4
	F1528L		2				2
	I1533V		2				2
	I1533T	2					2
	F1543L	1	1				2
	I1544V	2				1	3
	I1548V	1				1	2
	E1553G		9	1			10
	G1581D	1	1				2
	K1584E		1			1	2

## Data Availability

The data supporting the conclusions of this article are provided within the article. The original datasets analyzed in this study are available from the corresponding author upon request. The nucleotide sequences reported in this study have been deposited in the GenBank database under the accession numbers OL338546–OL338939 for DII-S6 and OL338940–OL339333 for DIII-S6.
